# Postembryonic RNAi in *Heterorhabditis bacteriophora*: a nematode insect parasite and host for insect pathogenic symbionts

**DOI:** 10.1186/1471-213X-7-101

**Published:** 2007-09-05

**Authors:** Todd A Ciche, Paul W Sternberg

**Affiliations:** 1 Department of Microbiology and Molecular Genetics, Michigan State University, 2215 Biomedical Physical Sciences Building, East Lansing, MI 48824, USA; 2 Mail Code 156-29, California Institute of Technology, 1200 E. California Blvd., Pasadena, CA 91125, USA

## Abstract

**Background:**

*Heterorhabditis bacteriophora *is applied throughout the world for the biological control of insects and is an animal model to study interspecies interactions, e.g. mutualism, parasitism and vector-borne disease. *H. bacteriophora *nematodes are mutually associated with the insect pathogen, *Photorhabdus luminescens*. The developmentally arrested infective juvenile (IJ) stage nematode (vector) specifically transmits *Photorhabdus luminescens *bacteria (pathogen) in its gut mucosa to the haemocoel of insects (host). The nematode vector and pathogen alone are not known to cause insect disease. RNA interference is an excellent reverse genetic tool to study gene function in *C. elegans*, and it would be useful in *H. bacteriophora *to exploit the *H. bacteriophora *genome project, currently in progress.

**Results:**

Soaking L1 stage *H. bacteriophora *with seven dsRNAs of genes whose *C. elegans *orthologs had severe RNAi phenotypes resulted in highly penetrant and obvious developmental and reproductive abnormalities. The efficacy of postembryonic double strand RNA interference (RNAi) was evident by abnormal gonad morphology and sterility of adult *H. bacteriophora *and *C. elegans *presumable due to defects in germ cell proliferation and gonad development. The penetrance of RNAi phenotypes in *H. bacteriophora *was high for five genes (87–100%; *Hba-cct-2*, *Hba-daf-21*, *Hba-icd-1*; *Hba-nol-5*, and *Hba-W01G7.3*) and moderate for two genes (usually 30–50%; *Hba-rack-1 *and *Hba-arf-1*). RNAi of three additional *C. elegans *orthologs for which RNAi phenotypes were not previously detected in *C. elegans*, also did not result in any apparent phenotypes in *H. bacteriophora*. Specific and severe reduction in transcript levels in RNAi treated L1s was determined by quantitative real-time RT-PCR. These results suggest that postembryonic RNAi by soaking is potent and specific.

**Conclusion:**

Although RNAi is conserved in animals and plants, RNAi using long dsRNA is not. These results demonstrate that RNAi can be used effectively in *H. bacteriophora *and can be applied for analyses of nematode genes involved in symbiosis and parasitism. It is likely that RNAi will be an important tool for functional genomics utilizing the high quality draft *H. bacteriophora *genome sequence.

## Background

*Heterorhabditis bacteriophora *is a rhabditid entomopathogenic nematode (EPN) symbiotic with the enteric bacterium *Photorhabdus luminescens*, a dangerous liaison lethal to many insect hosts [1]. EPNs are applied globally for the biological control of insects. The nematode is also a potentially powerful animal model to study interspecies interactions such as mutualism, parasitism, and vector-borne disease. Genomics and genetics are available for the symbiont, and because it is a close relative to *Caenorhabditis elegans*, are being developed for the nematode. In addition, well-studied arthropod models (e.g. *Drosophila melanogaster*) can be used as hosts [2].

**Figure 1:**
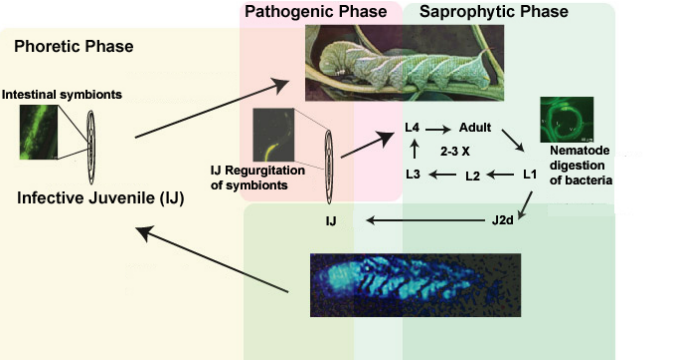
**Life-cycle of *Heterorhabditis bacteriophora *(adapted from [1])**. The non-feeding developmentally arrested dauer or infective juvenile (IJ) transmits a monoculture of symbiotic *P. luminescens *(GFP-labelled *P. luminescens *are shown) to the haemocoel of an insect host, where it regurgitates the bacteria. The bacteria rapidly kill the insect (usually <48) and grow to high densities allowing nematode growth and reproduction (The lower panel is an image taken from the bioluminescence of the bacteria). The nematodes grow for 2–3 generations, feeding on *P. luminescence*, after which (~10 d at 28°C) IJs are formed en masse, most transmitting symbiotic *P. luminescens*.

The developmentally arrested infective juvenile (IJ) nematodes exist in soil and transmit an average of 130 *P. luminescens *bacteria in their gut mucosa, sometimes for several months, before locating and infecting a suitable insect host [3]. The IJs search, locate and penetrate the insect exoskeleton or intestine, sometimes by using a buccal tooth, and then regurgitate the bacteria into the haemocoel (Figure 1). The association is an obligate mutualism since both the nematode and bacterium are required for insect pathogenicity.

The nematode is dependent on symbiotic bacteria for insect pathogenicity and nematode growth and reproduction since axenic IJs do not cause insect mortality nor develop and reproduce normally [4]. In contrast, *P. luminescens *are highly virulent when injected into the insect haemocoel, having an LD_50 _<30 cells for many insect hosts, but are dependent on the IJs for transmission and persistence outside of insect hosts. After the bacteria are regurgitated by the IJs [3], insect mortality rapidly ensues (usually <48 h) and the bacteria grow to high cell densities and produce insecticidal toxins, secondary metabolites to inhibit saprophytic microbes, bacteriophagous nematodes and savaging insects. In addition, the bacteria are required for nematode growth and reproduction [4,5].

Reproduction of *H. bacteriophora *is heterogonic: both automictic hermaphrodites and amphimictic females and males are formed [6]. The IJs are hermaphroditic, but generate automictic and amphimictic progeny that grow and reproduce for several generations on *P. luminescens*, after which, the hermaphroditic IJ stage is formed. The IJs are specifically colonized by *P. luminescens *and transmit the bacteria to new insect hosts.

*Heterorhabditis bacteriophora *is closely related to *C. elegans *(Rhabditidae) and can be propagated outside of insect hosts on agar based media and in liquid culture provided that a monoculture of symbiotic *P. luminescens *is present. We sought to further utilize the techniques and knowledge of *C. elegans *to study the interactions between *H. bacteriophora, P. luminescens *and insects.

One of these techniques is targeted gene silencing by RNAi, a powerful molecular genetic tool to elucidate gene function. In *C. elegans*, RNAi was originally performed by injecting dsRNA into the body of L4 animals [7,8] and also shown to be effective by soaking [9,10], on lawns of bacteria expressing dsRNA [11] or by expressing dsRNA in *C. elegans *cells [12]. RNAi by feeding [13,14], soaking [15] and injection [16] are amenable to high throughput methodologies. The dependence of *H. bacteriophora *for symbiotic bacteria for growth and reproduction makes RNAi by feeding problematic. Therefore, we developed a soaking protocol for efficient RNAi in *H. bacteriophora*. Using this protocol we detected highly penetrant and obvious phenotypes in *H. bacteriophora *for seven *C. elegans *orthologs, previously reported to have highly penetrant and obvious phenotypes in *C. elegans*. This is the first demonstration of RNAi by soaking in *H. bacteriophora*, a technique difficult for many nematodes other than *C. elegans *perhaps due to inefficient uptake of environmental dsRNA [[17], M. Montgomery pers. comm.]. This technique is amenable to high-throughput methodologies and should greatly enhance the analysis of gene function in *H. bacteriophora*.

## Results and discussion

### Optimization of RNAi methodology for *H. bacteriophora*

Since *H. bacteriophora *requires *P. luminescens *for growth and reproduction, one strategy for RNAi might be to express *H. bacteriophora *dsRNA in *Escherichia coli *HT115 (DE) delivered while in co-culture with *P. luminescens*. We tested the validity of this approach by determining if highly penetrant ds *Cel*-*pop-1 *RNA, retains silencing activity in *C. elegans *when grown in co-cultures of *P. luminescens. C. elegans *L4 grown on lawns of HT115 expressing ds *Cel-pop-1*RNA resulted in embryonic lethality (Emb) with 100% penetrance. However, silencing was abolished when as little as 2% *P. luminescens *were added (0% Emb) and to a lesser extent in the presence of 2% *Escherichia coli *OP50 (data not shown). Therefore, the strategy to deliver dsRNA as a HT110-*P. luminescens *co-culture was not feasible, presumably due to nucleases produced by *P. luminescens*. We then attempted to use a soaking methodology to deliver dsRNA for RNAi in *H. bacteriophora*.

Soaking L1 *H. bacteriophora *in M9 buffer resulted in high lethality (>90%). Survival of L1 larvae was improved in S-basal media and found to be optimal in Ringer's solution buffered with HEPES (pH 6.9). Initial experiments soaking L1 larvae for 24 h in Ringer's solution sometimes resulted in significant adult sterility. However, using L1s harvested from pure monocultures of *P. luminescens*, the adult nematodes were fertile. Thus, a pure monoxenic culture of *H. bacteriophora-P. luminescens *is important for survival and development of the nematodes during and after RNAi.

From 850 EST sequences, kindly communicated by Ann Burnell (National University of Ireland, Maynooth, Ireland), seven orthologs that had obvious and highly penetrant RNAi phenotypes in *C. elegans*, were chosen for RNAi by soaking in *H. bacteriophora *(Table 1, see additional file 1). Three other ESTs whose *C. elegans *orthologs had no discernable RNAi phenotype in *C. elegans *were also tested.

**Table 1:** Candidate *H. bacteriophora *genes for RNAi gene silencing

**Gene^A^**	**Predicted Function^B^**	***C. e*. Ortholog**	**%Identity, length^C^**	***C. elegans *Phenotypes^D ^[ref]**
*Hba-cct-2*	T-complex chaperonin	T21B10.1 (*Cel-cct-2*)	79, 357	Emb, Ste [13,14], Pch, Pvl, [14], Lva [13]
*Hba-daf-21*	Hsp-90 chaperonin	C47E8.5 (*Cel-daf-21*)	79, 804	Emb, Ste [31]
*Hba-icd-1*	BTF3 transcription factor Anti-apoptotic	C56C10.8 (*Cel-icd-1*)	75, 529	Emb [13,14, 31], Gro [13,14,15], Stp [14,15], Bmd, Clr, Sck [13], Unc [14]
*Hba-nol-5*	Ribosome biogenesis Nop58p/Nop5p	W01B11.3 (*Cel-nol-5*)	74, 634	Lva [14,31,53], Emb, Lva [31,53], Gro, Muv, Pvl, Sle, Stp, [31]
*Hba-W01G7.3*	RNA polymerase subunit L	W01G7.3 (*Cel-W01G7.3*)	80, 81	Emb [13,14,15,31], Gro [14,31], Ste, Stp [31], Pvl [14], Lva [15]
*Hba-rack-1*	G protein β-like subunit	K04D7.1 (*Cel-rack-1*)	75, 441	Gro [13,14], Emb, Egl [13], Slu [14]
*Hba-arf-1*	ADP-ribosylating factor	B0336.2 (*Cel-arf-1*)	75, 219	Emb [13,14,36], Unc [13,14], Pvl, Rup, Ste [13], Gro [14]
*Hba-ben-1*	β-tubulin, benomyl sensitivity	C54C6.2 (*Cel-ben-1*)	78, 518	no phenotype reported
*Hba-mrp-4*	Mrp-1 multidrug resistance family	F21G4.2 (*Cel-mrp-4*)	61, 468	no phenotype reported
*Hba-nhr-47*	nuclear hormone receptor	C24G66.4 (*Cel-nhr-47*)	65, 269	no phenotype reported

^A ^Based on ESTs, see methods for accession #'s.

^B ^Based on Wormbase gene description for *C. elegans *ortholog.

^C ^Based on BLASTN analysis in Wormbase.

^D ^RNAi phenotypes reported previously. Abbreviations are: Bmd = body morphology defect, Clr = clear, Egl = egg laying defective, Emb = embryonic lethal, Gro = slow growth, Muv = multivulva, Pch = patchy coloration, Pvl = protruded vulva, Sck = sick, Sle = slow embryonic development, Slu = sluggish, Ste = sterile, Stp = sterile progeny, Unc = uncoordinated

**Figure 2:**
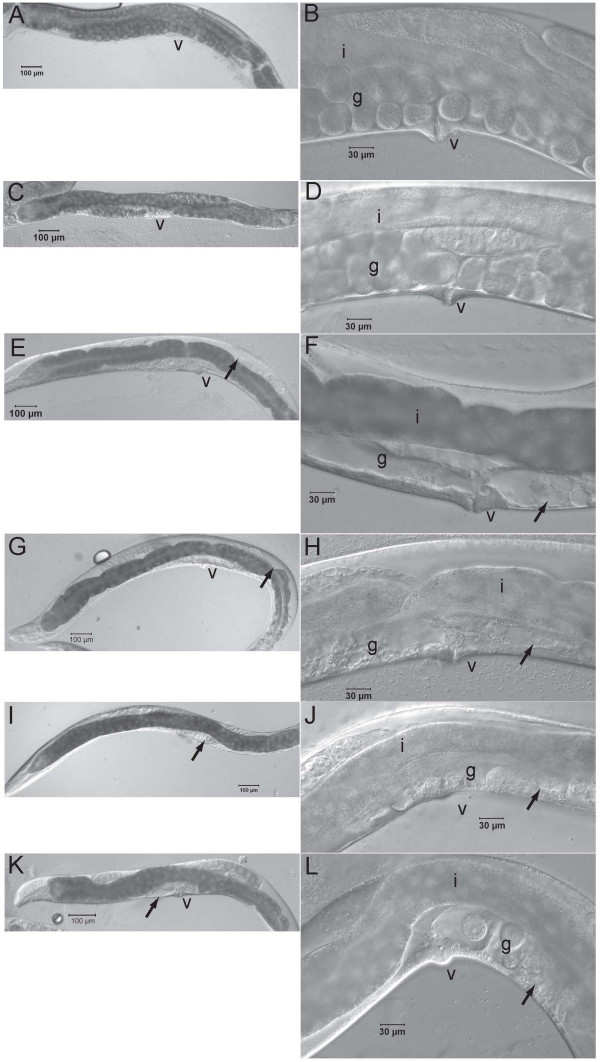
**Postembryonic RNAi phenotypes of *H. bacteriophora***. *Heterorhabditis bacteriophora *adult hermaphrodites 80–96 h after soaking L1s with A., B. no dsRNA added. C., D. ds *Hba-ben-1 *RNA, E., F. ds *Hba-cct-2 *RNA, G., H. ds *Hba-daf-21 *RNA, I., J. ds *Hba-icd-1 *RNA, and K., L. ds *Hba-nol-5 *RNA. v = vulva, i = intestine, g = gonad, ab = abnormal gonad.

RNAi by soaking L1s in dsRNA corresponding to portions of *Hba-cct-2, Hba-icd-1, Hba-daf-21, Hba-nol-5*, *Hba*-*W01G7.3, Hba-K04D7.1 *and *Hba-arf-1 *were successful as evidenced by sterility, defective gonad development and germline proliferation in adult animals (Table 2, Figure 2). L1s soaked in Ringer's solution with no dsRNA added or dsRNA corresponding to portions of *Hba-mrp-4, Hba-ben-1*, and *Hba-nhr-47*, resulted in adults with normal fertility, gonad morphology and oocyte formation (Table 2, Figure 2).

**Table 2:** *H. bacteriophora *and *C. elegans *RNAi Phenotypes

	**Trial**					
	
**Nematode Gene (Phenotype)**	**1 %Pen.(n)^A^**	**2 %Pen.(n)**	**3 %Pen.(n)**	**4 %Pen.(n)**	**5 %Pen.(n)**	**6 %Pen.(n)**
***H. bacteriophora*^B^**						

(-)water (Ste)	0 (49)	0 (17)	0 (20)	0 (39)	0 (40)	0 (30)
*Hba-cct-2*	100 (34)	100 (36)	100 (16)	96 (24)	94 (64)	100 (34)
*Hba-daf-21*	100 (36)	100 (19)	100 (30)	92 (24)	82 (34)	87 (47)
*Hba-icd-1*	100 (31)	100 (38)	87 (15)	100 (15)	98 (40)	98 (40)
*Hba-nol-5*	95 (39)	100 (32)	100 (41)	93 (30)	57 (28)	72 (32)
*Hba-W01G7.3*	95 (39)	100 (34)	95 (39)	100 (33)	83 (47)	86 (49)
*Hba-rack-1*	31 (39)	82 (22)	28 (25)	61 (28)	10 (39)	33 (33)
*Hba-arf-1*	50 (32)	67 (15)	47 (15)	nd^C^	nd	nd
*Hba-ben-1*	0 (48)	38 (21)	11 (36)	0 (45)	0 (42)	0 (33)
*Hba-nhr-47*	nd	12 (17)	3 (36)	nd	nd	nd
*Hba-mrp-4*	0 (57)	10 (21)	0 (26)	nd	nd	nd

***C. elegans*^D^**						

*Cel-cct-2 *(Pvl)	83 (86)	71(97)	53 (92)	nd	nd	nd
(Ste)	100 (86)	100 (97)	100 (92)	nd	nd	nd
*Cel-icd-1 *(Pvl)	81 (118)	86 (114)	nd	nd	nd	nd
(Ste/Stp)	100(118)	100 (114)	nd	nd	nd	nd
*Cel*-W01G7.3 (Pvl)	10 (94)	9 (64)	12 (69)	nd	nd	nd
(Ste)	100 (94)	100 (64)	<100 (>50)	nd	nd	nd
(Egl, Stp)	nd	nd	few	nd	nd	nd
*Cel-nol-5 *(Pvl)	68 (94)	64 (98)	nd	nd	nd	nd
(Ste)	100 (94)	100 (98)	nd	nd	nd	nd
*Cel*-*rack-1*(Pvl)	19 (66)	4 (80)	3 (72)	nd	nd	nd
(Ste)	100 (66)	<100(>50)	<100(>50)	nd	nd	nd
(Egl, Stp)	0	Few	few	nd	nd	nd
*Cel-ben-1*	wt^E ^(>50)	wt (>50)	wt (>50)	nd	nd	nd

^A ^Percent penetrance (% Pen.), number of worms examined (n).

^B ^Number of sterile adult female or hermaphrodites with defective gonads from RNAi by soaking, see methods for details.

^C ^Not determined

^D ^Postembryonic phenotypes from RNAi by feeding, see methods for details.

^E ^Indistinguishable from wt.

### RNAi of *H. bacteriophora*

The *C. elegans cct-2 *(T21B10.1) gene encodes a component of eukaryotic T-complex chaperonin (CCT or TRiC)[18]. CCT is a group II chaperonin of similar structure to the prokaryotic GroEL chaperonin, but found only in Eukarya and Archea [19]. CCT/TRiC is required to fold actin [20], tubulin [21], cyclin E1 [22] and also 10% of newly synthesized cytoplasmic proteins [23], including proteins with WD40 domains [24]. In *C. elegans, mec-3 *independent expression of *cct-2 *touch receptors has also been demonstrated, suggesting a role for CCT in touch receptor function, possibly through microtubule assembly [25].

In *C. elegans*, published RNAi experiments of *cct-2 *resulted in embryonic lethal (Emb) and sterile (Ste) phenotypes [13,14] and sometimes a protruding vulva (Pvl)(Table 1)[14]. RNAi by feeding L1 *C. elegans *with bacteria expressing ds *Cel-cct-2 *RNA resulted in a highly penetrant Ste phenotype with Pvl also observed (Table 2). Soaking L1 *H. bacteriophora *in ds *Hba-cct-2 *RNA resulted in a highly penetrant (100% in 4 trials, 96% and 94% in two other trials) Ste phenotype (Table 2). The cause of sterility was likely due to defective gonad development and absence of germ cells (Figure 2E, F). Mature *H. bacteriophora *hermaphrodites which lack gonads have an empty or transparent appearance due to a void space in the pseudocoelom normally occupied by the gonad (Figure 2E, F). Both *C. elegans *and *H. bacteriophora *treated with their respective ds *cct-2 *RNA were sterile and had abnormal gonads, although only *C. elegans *had a protruded vulva (Table 2). The comparable penetrances of these phenotypes suggest that RNAi by soaking in *H. bacteriophora *is nearly as efficient as by feeding in *C. elegans*.

The *C. elegans daf-21*(C47E8.5) gene encodes a member of the Hsp90 family of molecular chaperones important for maturation of signal transduction kinases in neurons involved in odorant perception and required for larval development [26]. Hsp90 is also expressed in the gonad and required for germline development [27], but is also expressed somatically during stress as part of the *age-1 *related aging regulon [28-30]. A published RNAi experiment of *daf-21 *in *C. elegans *resulted in Emb and Ste phenotypes [31]. The post-embryonic phenotype of *H. bacteriophora *treated with ds *Hba-daf-21 *RNA resulted in sterile animals with abnormal gonad morphology (Figure 2G, H). Like RNAi of *Hba-cct-2*, RNAi of *Hba-daf-21 *was highly penetrant (Table 2, 100% for 3 trials and 92%, 82%, 87% each for single trials).

**Figure 3:**
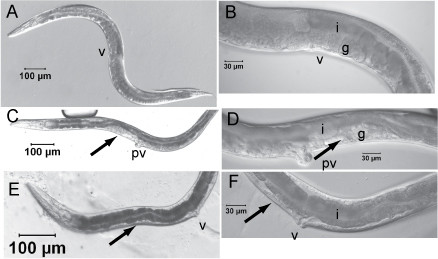
**Postembryonic RNAi phenotypes of *C. elegans***. *Caenorhabditis elegans a*dult hermaphrodites of 72–80 h after L1s were placed on *E. coli *HT110 expressing A., B. dsGFP RNA, C., D., ds *Cel-icd-1 *RNA, E., F., and ds *Cel-nol-5 *RNA. v = vulva, pv = protruding vulva, i = intestine, g = gonad. Arrow denotes abnormal gonad.

The *C. elegans icd-1 *(C56C10.8) gene encodes the beta subunit of nascent polypeptide associated complex (βNAC) associated with the mitochondrial membrane and an inhibitor of apoptosis [32]. Published RNAi experiments of *icd-1 *in *C. elegans *resulted in Emb, slow growth and Ste phenotypes (Table 1) [13-16]. We detected highly penetrant Ste, Pvl, and defective gonad morphology phenotypes by feeding *C. elegans *ds-*icd-1 *(Table 2, Figure 3). Postembryonic RNAi of *H. bacteriophora *resulted in Ste phenotype due to an abnormal gonad with little germ cell proliferation (Figure 2I, J). This phenotype was highly penetrant (Table 2, 100% Ste in 3 trials, 98% in 2 trials, and 87% in one trial). We did not observe apoptosis in *H. bacteriophora*, and we were unable to generate sufficient males to observe defects in male tale rays, phenotypes observed in *C. elegans *[32].

The *C. elegans *W01B11.3 gene, recently named *nol-5 *(Jonathan Hodgkin, personal communication), is predicted to encode an ortholog of the ribosome biogenesis protein Nop58p/Nop5p and is likely essential for information processing. Published RNAi experiments of *Cel-nol-5 *usually resulted in larval arrest and Emb phenotypes, with multivulva, protruding vulva, slow growth, maternal sterility and sterile progeny phenotypes also observed [14,31]. RNAi by feeding ds *Cel-nol-5 *RNA resulted in Pvl, Ste with abnormal gonad development phenotypes (Table 2, Figure 3E, F). RNAi of *Hba-nol-5 *in *H. bacteriophora *resulted in a Ste phenotype with abnormal gonads (Figure 2K, L), which was highly penetrant (Table 2, 100% two trials, 95%, 93%, 72% and 57% in four other trials).

The *C. elegans *gene W01G7.3, like *Cel-nol-5*, is likely essential for information processing since it is predicted to encode subunit L of RNA polymerase. RNAi of W01G7.3 in *C. elegans *resulted in Emb phenotype with several other growth defects observed (Table 1) [13-15,31]. RNAi of *Hba-W01G7.3 *resulted in Ste phenotype with an abnormal gonad, likely due to the essential role for this protein in cell viability and division (Table 2). This phenotype was highly penetrant, with >83% of adults being sterile with abnormal gonads (Table 2).

The *C. elegans rack-1 *(K04D7.1) gene is predicted to encode the homolog of mammalian receptor of activated C kinase. Published RNAi experiments of *Cel-rack-1 *in *C. elegans *resulted in less obvious and penetrant phenotypes than those described above: Emb, slow growth, egg laying defect, aldicarb resistance, and larval lethality (Table 1)[13,14]. RNAi of *Hba-rack-1 *resulted in 10–82% of animals with a Ste phenotype and abnormal gonad development (Table 2, Figure 2). Thus, lower penetrances were observed using ds-*rack-1 *RNA for both *H. bacteriophora *and *C. elegans*.

The *C. elegans arf-1 *(B0336.2) gene encodes a predicted ADP-ribosylating factor (ARF). ARFs are known to regulate membrane trafficking and the actin cytoskeleton, phospholipase D1 and phosphatidylinositol 4-phosphate 5-kinase [33]. RNAi of *Cel-arf-1 *resulted in Emb and a variety of other defects (Table 1) [14,15]. RNAi of *Hba-arf-1 *resulted in a Ste phenotype with abnormal gonads in 50%, 67% and 47% in each of three trials (Table 2).

The *C. elegans ben-1 *(C54C6.2) gene, also known as *tbb-1*, encodes the beta subunit of tubulin, which when disrupted, results in resistance to benzimidizole [34]. RNAi of *ben-1 *in *C. elegans *usually resulted in no observed defect [13,35,36] although Emb, abnormal post-embryonic development, larval arrest and larval development were sometimes observed [13,35,36]. RNAi of *Hba-ben-1 *in *H. bacteriophora *usually resulted in no observable defect, although a Ste (38%, 11%) phenotype was observed in two experiments.

The *C. elegans mrp-4 *(F21G4.2) gene is predicted to encode a multidrug-resistance associated (*mrp-1 *type) protein related to human cystic fibrosis transmembrane conductance regulator, CFTR. RNAi of *mrp-4 *in *C. elegans *resulted in no observed phenotype [13,16]. Since the *P. luminescens *symbiont of *H. bacteriophora *produces several known secondary metabolites, such as hydroxystilbene antibiotics, anthraquinone pigments [37] and a photobactin siderophore [38], it is possible that *H. bacteriophora *multidrug-resistance associated proteins might be involved in exporting these secondary metabolites. RNAi of *Hba-mrp-4 *in *H. bacteriophora *resulted in no observed defect in two trials and 10% sterility in one trial (Table 2). It is likely that *H. bacteriophora*, like *C. elegans *[39], has several multidrug-resistance associated proteins and this redundancy might be responsible for the weak phenotype from RNAi of *Hba-mrp-4*. Alternatively, other resistance mechanism might be employed to resist secondary metabolites produced by *P. luminescens*.

The *C. elegans nhr-47 *(C24G66.4) gene is predicted to encode a nuclear hormone receptor and is induced upon worm exposure to estradiol [40]. Published RNAi experiments of *Cel-nhr-47 *in *C. elegans *resulted in no observed phenotype [13,16,35]. Similarly, RNAi of *Hba-nhr-47 *in *H. bacteriophora *usually resulted in no observed phenotype, although 12% and 3% Ste animals were observed (Table 2, Figure 2).

### Quantification of RNAi by Real-time RT-qPCR

To determine the extent of RNAi silencing in *H. bacteriophora*, quantitative real-time RT-qPCR experiments were performed. Message levels of *Hba-cct-2 *and *Hba-nol-5 *were quantified relative to *Hba-ben-1 *when treated with the specific or unspecific dsRNA (i.e. for *Hba-cct-2 *and *Hba-nol-5*, RNAi using ds *Hba-nol-5 *and ds *Hba-cct-2 *RNAs were used, respectively). Relative amounts of mRNA were determined using the ΔΔC_t _method. RNAi of *Hba-cct-2 *resulted in mRNA levels 5.8 × 10^-3 ^and 8.5 × 10^-6 ^relative to the nonspecific dsRNA control. RNAi of *Hba-nol-5 *resulted in mRNA levels 2.6 × 10^-3 ^and 7.1 × 10^-2 ^relative to the nonspecific dsRNA control. RNAi of *Hba-ben-1 *resulted in mRNA levels 1.6 × 10^-2 ^and 9.9 × 10^-4^relative to two nonspecific dsRNA controls: *Hba-nol-5 *in an *Hba-cct-2 *RNAi experiment or *Hba-cct-2 *in a *Hba-nol-5 *experiment, respectively. From these data we conclude that RNAi by soaking in *H. bacteriophora *is potent and specific.

### RNAi treated *H. bacteriophora *are normal in symbiotic host-bacterial interactions

One of our goals for developing RNAi in *H. bacteriophora *is to use this technique along with imminent high quality draft (6× coverage) *H. bacteriophora *genome sequence (R. Wilson, personal communication), to identify genes involved in symbiotic host-bacterial interactions. Many of the genes silenced have essential functions in the nematode and we sought to determine if these RNAi treated animals have defects in symbiotic host-bacterial interactions. RNAi treated worms for all 10 genes described above were reared on GFP-labeled *P. luminescens *and observed for bacterial colonization in the adult nematode intestine. No difference was seen in the presence of GFP-labeled *P. luminescens *in the intestines of RNAi treated and untreated worms (see additional file 2). This observation suggests that specific genes involved in symbiotic host-bacterial interactions can be identified using RNAi.

## Conclusion

Gene silencing by RNAi is a powerful reverse genetic tool to study gene function. Although, RNAi using long dsRNA is robust in *C. elegans*, it is inefficient in many other nematodes. The robustness of RNAi in *C. elegans *can be partially attributed to the systemic RNAi defective gene, *Cel-sid-1*, involved in the transport of long dsRNA [41,42] and recently, *Cel-sid-2 *required for efficient transport of dsRNA in the *C. elegans *intestine [17]. Recently, the systemic RNAi defective gene, *Hba-sid-1*, was identified in *H. bacteriophora *[43] which might partially explain the obvious phenotypes and high penetrances of RNAi in *H. bacteriophora*. Because the nematode egg yolk is synthesized by intestinal cells, it is possible that the Ste and gonad defective phenotypes are caused by RNAi of only the nematode intestinal cells. However, the low amounts of transcript levels detected in L1s following RNAi suggests that RNAi is nearly systemic. This hypothesis could be tested, in principle, by performing RNAi on muscle tissue, for example by silencing the *unc-22 *gene conferring a twitching phenotype to *C. elegans *[44]. Attempts to use degenerate primers to amplify *Hba-unc-22 *were not successful and *Hba-unc-22 *has not been found in existing *H. bacteriophora *EST datasets. After the completion of the *H. bacteriophora *genome, this and many other RNAi experiments will be performed.

This study demonstrates that RNAi by soaking is an efficient methodology for gene silencing in *H. bacteriophora*, which can be applied to study many aspects of the unique biology including parasitism and mutualism. One limitation to this methodology is the development of RNAi treated nematodes to adults versus the infective juvenile stage, the later which transmits the symbiotic bacteria and are insect parasitic. The lack of IJ development from RNAi treated larvae is likely due to the large amount of food signal provided by the confluent lawn of *P. luminescens *on which the L1s are placed. The density of L1 larvae might also influence the development of the L1 larvae to the IJ stage. The severe and highly penetrant phenotypes observed here suggests that RNAi will be a useful tool to study gene function in *H. bacteriophora*, i.e. related to symbiont transmission, parasitism, sex determination, stress resistance and infective juvenile formation.

More generally, mixed results concerning the efficiency of RNAi have been reported in several clades of parasitic nematodes [45]. Problems encountered using RNAi in parasitic nematodes includes non-target effects of dsRNA, variable efficiencies of RNAi with regard to the target gene silenced and dsRNA preparation, and *in vitro *cultivation. These problems appear to be less of a concern for *H. bacteriophora *due to the high specificity and penetrances of RNAi. In the potato cyst nematode *Globodara padilla*, RNAi of neuronal FMRF amide-like peptides was surprisingly robust [46]. The results reported here are promising to the applicability of RNAi to study gene function in *H. bacteriophora*. However, further refinements of the RNAi methodology are expected when targeting diverse genes expressed in different tissues, when applied to nematodes of different developmental stages and when applied to larger gene sets.

## Methods

### Strains and growth conditions

*Heterorhabditis bacteriophora *strain TTO1 was kindly provided by Ann Burnell (National University of Ireland-Maynooth). *Photorhabdus luminescens *subsp. *laumondii *were isolated from IJs that were surfaced sterilized for 5 min in 2% commercial bleach (~ 12% sodium hypochlorite) and allowed to release the bacteria on 2% Proteose Peptone #3 (PP3)(Difco, Detroit, MI) agar. Glycerol stocks (25% vol/vol sterile glycerol) of the bacterium were stored at -80°C. The nematodes were cryopreserved as described previously [47] and maintained by infecting Greater Waxmoth larvae, *Galleria mellonella *(Rainbow Mealworms, Compton, CA) or propagated on lawns of symbiotic bacteria as follows: the primary phase variant of *P. luminescens *was grown overnight at 28°C in 3 ml of PP3 after which 50 (MICRO) ul were spread on NA+chol (1.5× Nutrient Broth, 1.5% agar (Difco, Detroit, MI) and 10 μg/ml cholesterol), the inoculated plates were incubated at 28°C overnight, after which 50–100 surface sterilized IJs were added. The nematode cultures were grown at 28°C and eggs were collected ~ 86 h or IJs ~ 10 d after inoculation. An inbred line, M31e, was obtained by self-fertilizing the hermaphrodites for 13 generations by placing single IJs onto NA+chol containing a lawn of *P. luminescens *and inoculating new cultures with single IJ offspring, a process repeated 13 times.

*Caenorhabditis elegans *N2 were maintained on *E. coli *OP50 seeded NGM agar as previously described [48].

### Generation of dsRNA

A dataset of approximately 650 expressed sequence tags (ESTs) obtained from *H. bacteriophora *HP88 IJs recovered on lawns of symbiotic *P. luminescens *was kindly provided by Ann Burnell. The EST dataset was analyzed for the presence of *C. elegans *orthologs by BLASTX analysis [49] and for gene function and RNAi phenotypes using gene annotations and WormBase [50]. Seven ESTs were chosen that had severe RNAi phenotypes in *C. elegans *(*Hba-cct-2*, *Hba-icd-1*, *Hba-daf-21*, *Hba-nol-5*, *Hba-W01G7.3, Hba-K04D7.1, Hba-arf-1*) and one potentially involved in symbiosis (*Hba-mrp-4*), potentially conferring resistance to benomyl (*Hba-ben-1*) and a nuclear hormone receptor (*Hba-nhr-47*) (Table 1, see additional file 1). Oligonucleotide primers were designed using the EST sequence and Primer3 [51]. Large introns were avoided by aligning the EST sequence with the *C. elegans *genome and using the gene structure content of WormBase. The T7 RNA polymerase promoter sequence taatacgactcactatagggaga (T7) was added to each of the 5' ends of the PCR primers for in vitro transcription to generate dsRNA. The primer sequences are: *Hba*-*cct-2*T7f, (T7)cagccaaagaggatggagaa; *Hba*-*cct-2*T7r, cctccgagaacaagtgcaag; *Hba*-*daf-21*T7f, (T7)cgagaaattgccgaagata; *Hba*-*daf-21*T7r, (T7)tggcaactccagaccttctt; *Hba-icd*-*1*T7f, (T7)agggaactccacggagaaag; *Hba-icd-1*T7r, (T7)tcggcctttgtctcattctt; *Hba-nol-5*T7f, (T7)ggagctagagcagccatacg; *Hba-nol-5*T7r, (T7)tgtgcaggctgtatcacttc; *Hba*-*W01G7.3*T7f, (T7)aagttcggccatcaaatcag; *Hba-W01G7.3*T7r, (T7)caaaggttcctaacgctgct; *Hba*-*K04D7.1*T7f, (T7)ggacaattcgctctttctgg; *Hba*-*K04D7.1*T7r, (T7)agcgatccatcaggtgaaac; *Hba-arf-1*T7f, aaactgggcgaaatcgttact; *Hba-arf-1*T7r, ggcagcattcatagcattagg; *Hba-ben-1*T7f, (T7)aaatggcggcaagtatgttc; *Hba-ben-1*T7r, (T7)gaaggaacgacggaaaatga; *Hba-mrp-4*T7f, (T7)cggtcgagagtcaatacaagg; *Hba-mrp-4*T7r, (T7)gccggggtaatgtttgaatg; *Hba-nhr-47*T7f, (T7)cgatgcagctagtcaacgaa; *Hba-nhr-47*T7r, (T7)ggcctaattcctaacgcagtc.

Genomic DNA was purified from *H. bacteriophora *IJs collected from *P. luminescens *containing NA+chol plates using a modified CTAB/phenol extraction protocol [52] after which bacterial DNA was digested with DpnI (New England Biolabs, Bedford, MA). 100–200 ng of template was added to a PCR reaction containing 20 pM of each primer, in a 50 μl vol using the standard reaction provided for Taq (Promega, Madison, WI). The PCR condition used was: 1.94°C for 3 min, 2.94°C 45 sec, 3. 57°C 30 sec, 4. 72°C 45 sec, 5. repeat steps 2–4 30× then 6.72°C 10 min. The PCR reactions were analyzed for a single band of predicted size on a 1.2% agarose gel. 5 μl of the PCR reaction was then used directly for *in vitro *transcription using the Megascribe T7 kit (Ambion, Austin, TX) or T7 RiboMax (Promega) according to the instructions provided, except the transcription reactions were incubated for >6 h at 37°C. The DNA templates were removed by DNAse treatment and then dsRNA precipitated by adding 1/10 vol. of 5 M ammonium acetate and 2.5 vol. of 100% ethanol for >1 h at 4°C. The precipitated dsRNA was centrifuged for 30 min at 16,000 × *g *and then washed with 70% ethanol prepared in RNAse free water. After air drying for 5 min, the pellet was dissolved in 25 μl of RNAse free water. The quality of the transcribed RNA was determined by running 1 μl on a 1.2% agarose gel and quantified (A_260_) using a NanoDrop (Nanodrop Technologies, Wilmington, DE).

### RNAi of *H. bacteriophora*

*Heterorhabditis bacteriophora *eggs were harvested from NA +chol. containing *P. luminescens *usually 82–86 h after the addition of 50–100 IJs when grown at 28–29°C. Washing 1–2 week old IJs three times in 15 ml of Ringer's solution (100 mM NaCl, 1.8 mM KCl, 2 mM CaCl_2_, 1 mM MgCl_2_, 5 mM HEPES pH 6.9) improved the synchrony of IJ recovery. At this time most of the eggs were at the pretzel stage of embryonic development. The eggs were harvested by washing the plates 3× with 2 ml of sterile Ringer's solution and bacteria removed by filtering on a 10 μm TCTP membrane (Millipore, Billerica, MA) with a gentle vacuum applied. Eggs and adult hermaphrodites were washed 3 times with 10 ml of Ringer's solution after which eggs were purified from hermaphrodites by their different settling rates. The eggs were concentrated by centrifugation, 2,000 × g for 1–2 min and resuspended in Ringer's to a concentration of ~ 5 eggs per μl. 4 μl of 5–7.5 mg/ml dsRNA was then added to eggs at total volume of 20 μl. The eggs hatch in the dsRNA solution while they are incubated for >24 h at 28°C. The resulting L1s were then placed on 18–24 h lawns of *P. luminescens *on NA+chol plates. Postembryonic abnormalities caused by the dsRNA were observed 2–5 days post RNAi treatment.

### Quantification of RNAi by Real-time RT-qPCR

RNAi was performed as described above except 150–250 L1s were soaked with 4 ul of 5–7.5 mg/ml dsRNA in a total volume of 15 μl. After 24 h, the L1s were washed 3× in Ringer's solution. The RNA was extracted by adding 500 ul Trizol (Invitrogen, Carlsbad, CA) and stored at -80°C before extracting according to the manufacturer's instructions. 80–100 ng total RNA treated with 10 U DNAse I for 15 min at 37°C. The following primers were used for RT-qPCR where one of the primers lies outside the dsRNA used for RNAi: *Hba-nol-5*RTfor: gtgagatcagtcgagcacca, *Hba-nol-5*RTrev: cggaggagatcgagtcaaag, *Hba-ben-1*RTfor: tcatttcggatgaacatgga, *Hba-ben-1 *RTrev: ggacggaatagcagtccaaa, *Hba-cct-2 *RTfor: acttcctggtatgtatcagcc, *Hba-cct-2 *RTrev: gccataactccagcatccgc. 50 ng of total RNA was reverse transcribed using, ThermoScript Reverse Transcriptase (Invitrogen) according to the Manufacturer's instruction, 56°C annealing temperature, using antisense primers. Real-time RT-qPCR was performed according to the manufacturer's instructions using Syber Green PCR Master Mix (Applied Biosystems Incorporated, Foster City, CA) performed using a ABI Prism 7900HT Sequence Detection System located at the Michigan State University Research Technology Support Facility. Using *Hba-ben-1 *as an internal standard, *Hba-cct-2 *and *Hba-nol-5 *mRNAs were quantified either in RNAi experiments using specific dsRNA or the unspecific dsRNA corresponding to the other gene (i.e. *Hba-cct-2 *mRNA treated with ds *Hba-nol-5 *RNA). Non-RT samples were used as negative controls. The experiments were repeated twice.

### RNAi by feeding of *C. elegans*

RNAi by feeding was done as published [13], except that eggs were harvested by alkaline hypochlorite lysis of gravid hermaphrodites and added to the HT115 expressing dsRNA. The following HT115(DE3) clones expressing dsRNA in the feeding vector L440 were obtained from the Ahringer RNAi feeding library : *pop-1*, I-1K04; *cct-2*, II-6O12; *icd-1*, II-5I03; *arf-1*, III-3A13; *nol-5*, I-1O18; W01G7.3, II-9A23; K04D7.1, IV-5K04; *ben-1*, III-1F10; *mrp-4*, X-1E23. Sterility of adults, abnormal gonad development and protruding vulva phenotypes were scored 72–80 h after eggs were placed on dsRNA expressing bacteria.

### Accession numbers

The accession numbers (Genbank, dbEST) for *H. bacteriophora *ESTs used in this study are: EE724174 (*Hba-cct-2*); EE724171–EE724173 (*Hba-daf-21*); EE724162, EE724228 (*Hba-icd-1*); EE724163–EE724164 (*Hba-nol-5*); EE724167–EE724168 (*Hba-W01G7.3*); EE724169–EE724170 (*Hba-arf-1*); EE724158–EE724161, EE724188, EE724199, EE7241206–EE7241209 (*Hba-rack-1*); EE724165–EE724166 (*Hba-ben-1*); EE724176 (*Hba-mrp-4*); EE724175 (*Hba-nhr-47*).

## Authors' contributions

T.A. Ciche designed and performed all experiments and wrote the paper. P.W. Sternberg mentored T.A. Ciche during these experiments by providing valuable advice, laboratory space and resources, and edited the paper. Both authors have read and approved the final version.

## Supplementary Material

Additional file 1 Alignment of *H. bacteriophora *ESTs to *C. elegans *genes. Alignments of ESTs used for RNAi to homologs in *C. elegans*. Bar in A = 300 bp for A-H and 600 bp for I and J. The panels are genome views from BLASTN analysis of *H. bacteriophora *ESTs (lower sequences) to the *C. elegans *genes (upper sequences) using Wormbase [50]. Blocks and lines indicate exons and introns, respectively. A. *Hba*-W01G7.3 B. *Hba-icd-1 *(C56C10.8.2) C. *Hba-rack-1 *(K04D7.1.1), D. *Hba-nol-5 *(W01B11.3), E. *Hba-arf-1 *(B0336.2.1), F. *Hba-ben-1 *(K01G5.7.1), G. *Hba-cct-2 *(T21B10.7.1), H. *Hba-daf-21 *(C47E8.5.2), I. *Hba-mrp-4 *(F21G4.2)., J. *Hba-nhr-47 *(C24G6.4.1). Click here for file

Additional file 2 GFP-labeled *P. luminescens *in *H. bacteriophora *adult hermaphrodites. *H. bacteriophora *adult hermaphrodites 80–96 h after soaking L1s and grown on lawns of GFP-labeled *P. luminescens *A., B., no dsRNA added, C., D., ds *Hba-cct-2 *RNA added. i = intestine. Click here for file
