# Supplementary figures and images for: Postembryonic RNAi in Heterorhabditis bacteriophora: a nematode insect parasite and host for insect pathogenic symbionts
Source: BMC Dev Biol. 2007 Sep 5;7:101. doi: 10.1186/1471-213X-7-101 (PMC2014770; doi:10.1186/1471-213X-7-101)

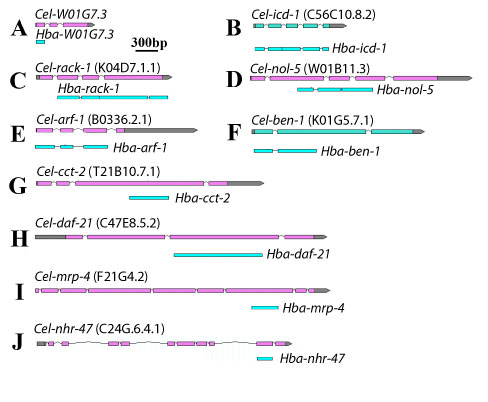

Supplement: Additional file 1 — Alignment of H. bacteriophora ESTs to C. elegans genes. Alignments of ESTs used for RNAi to homologs in C. elegans. Bar in A = 300 bp for A-H and 600 bp for I and J. The panels are genome views from BLASTN analysis of H. bacteriophora ESTs (lower sequences) to the C. elegans genes (upper sequences) using Wormbase [50]. Blocks and lines indicate exons and introns, respectively. A. Hba-W01G7.3 B. Hba-icd-1 (C56C10.8.2) C. Hba-rack-1 (K04D7.1.1), D. Hba-nol-5 (W01B11.3), E. Hba-arf-1 (B0336.2.1), F. Hba-ben-1 (K01G5.7.1), G. Hba-cct-2 (T21B10.7.1), H. Hba-daf-21 (C47E8.5.2), I. Hba-mrp-4 (F21G4.2)., J. Hba-nhr-47 (C24G6.4.1). [file 1471-213X-7-101-S1.jpeg]

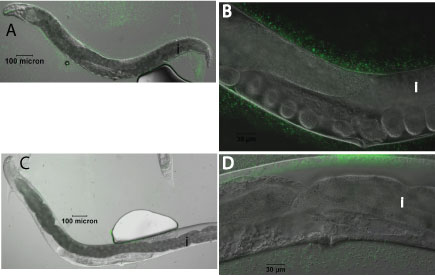

Supplement: Additional file 2 — GFP-labeled P. luminescens in H. bacteriophora adult hermaphrodites. H. bacteriophora adult hermaphrodites 80–96 h after soaking L1s and grown on lawns of GFP-labeled P. luminescens A., B., no dsRNA added, C., D., ds Hba-cct-2 RNA added. i = intestine. [file 1471-213X-7-101-S2.jpeg]
